# A lumped parameter modelling study of cerebral autoregulation in normal pressure hydrocephalus suggests the brain chooses to be ischemic

**DOI:** 10.1038/s41598-024-75214-6

**Published:** 2024-10-17

**Authors:** Grant Alexander Bateman, Alexander Robert Bateman

**Affiliations:** 1 Department of Medical Imaging, John Hunter Hospital, Locked Bag 1, Newcastle Region Mail Center, Newcastle, NSW 2310 Australia; 2 Newcastle University Faculty of Health, Callaghan Campus, Newcastle, NSW Australia; 3 School of Mechanical Engineering, University of New South Wales, Sydney, NSW Australia

**Keywords:** Autoregulation, Cerebral blood flow, Normal pressure hydrocephalus, Ischemia, CSF formation rate, Diseases, Pathogenesis, Engineering

## Abstract

Normal pressure hydrocephalus (NPH) is associated with a reduction in cerebral blood flow and an ischemic metabolic state. Ischemia should exhaust the available autoregulation in an attempt to correct the metabolic imbalance. There is evidence of some retained autoregulation reserve in NPH. The aim of this study is to model the cerebral autoregulation in NPH to discover a solution to this apparent paradox. A lumped parameter model was developed utilizing the known limits of autoregulation in man. The model was tested by predicting the cerebral blood volume changes which would be brought about by changes in resistance. NPH and the post shunt state were then modeled using the known constraints provided from the literature. The model successfully predicted the cerebral blood volume changes brought about by altering the cerebral perfusion pressure to the limit of autoregulation. The model suggests that NPH is associated with a balanced increase in resistance within the arterial and venous outflow segments. The arterial resistance decreased after modelling shunt insertion. The model suggests that the cerebral blood flow is actively limited in NPH by arteriolar constriction. This may occur to minimize the rise in ICP by reducing the apparent CSF formation rate.

## Introduction

The syndrome of normal pressure hydrocephalus (NPH) was first described by Adams et al. almost 60 years ago, in patients with a classical clinical triad of ataxia, incontinence and dementia^1^. These patients were found to have dilated ventricles but a normal CSF pressure^1^. It is well recognized that part of the syndrome of NPH involves a reduction in the cerebral blood flow (CBF). Global reductions in CBF within the brain have been measured to be between 14 and 28%, with the average reduction being approximately 20%^2–7^. A reduction in CBF of this magnitude should be associated with relative cerebral ischemia. Using a periventricular microdialysis catheter in NPH patients, there was a reduction in the periventricular glucose and an increase in lactate and pyruvate levels, indicating a reduction in energy metabolism^8^. There was also a significant increase in tissue oxygenation following CSF drainage^8^. In NPH, lactate is found within the lateral ventricles, indicating anerobic glycolysis must be occurring somewhere within the brain^9^. These findings of reduced energy metabolism would normally be expected to trigger dilatation of the arterioles to increase the brain’s perfusion and correct the metabolic imbalances found. However, this does not appear to occur in NPH. The possibilities for these findings could include that there is a fixed stenosis of the inflow vessels (i.e., they are unable to dilate) or that they are overwhelmed by compression of the venous outflow, secondary to the normal pressure hydrocephalus i.e. incapable of dilating further. However, despite this, there is evidence of some retained autoregulation reserve in NPH. In normal pressure hydrocephalus, in those who improved post shunting, the baseline hemispheric CBF was reduced by 28% but increased with acetazolamide testing by 53%, indicating some preserved autoregulation^7^. Similarly, Czosnyka et al. also found NPH was associated with preserved autoregulation^10^.

This raises an apparent paradox. The brain is ischemic in NPH and although the autoregulation is blunted, there is some preserved autoregulation reserve, but apparently the brain chooses not to utilize this available blood flow. This would indicate either some flaw in the autoregulation control, or another imperative may be overruling the need for an improved cerebral metabolism. The purpose of the current paper is to model the cerebral autoregulation of the brain in NPH and following shunt insertion, to try to answer why there is some preserved autoregulation which the brain apparently does not use.

## Results

**Fig. 1:**
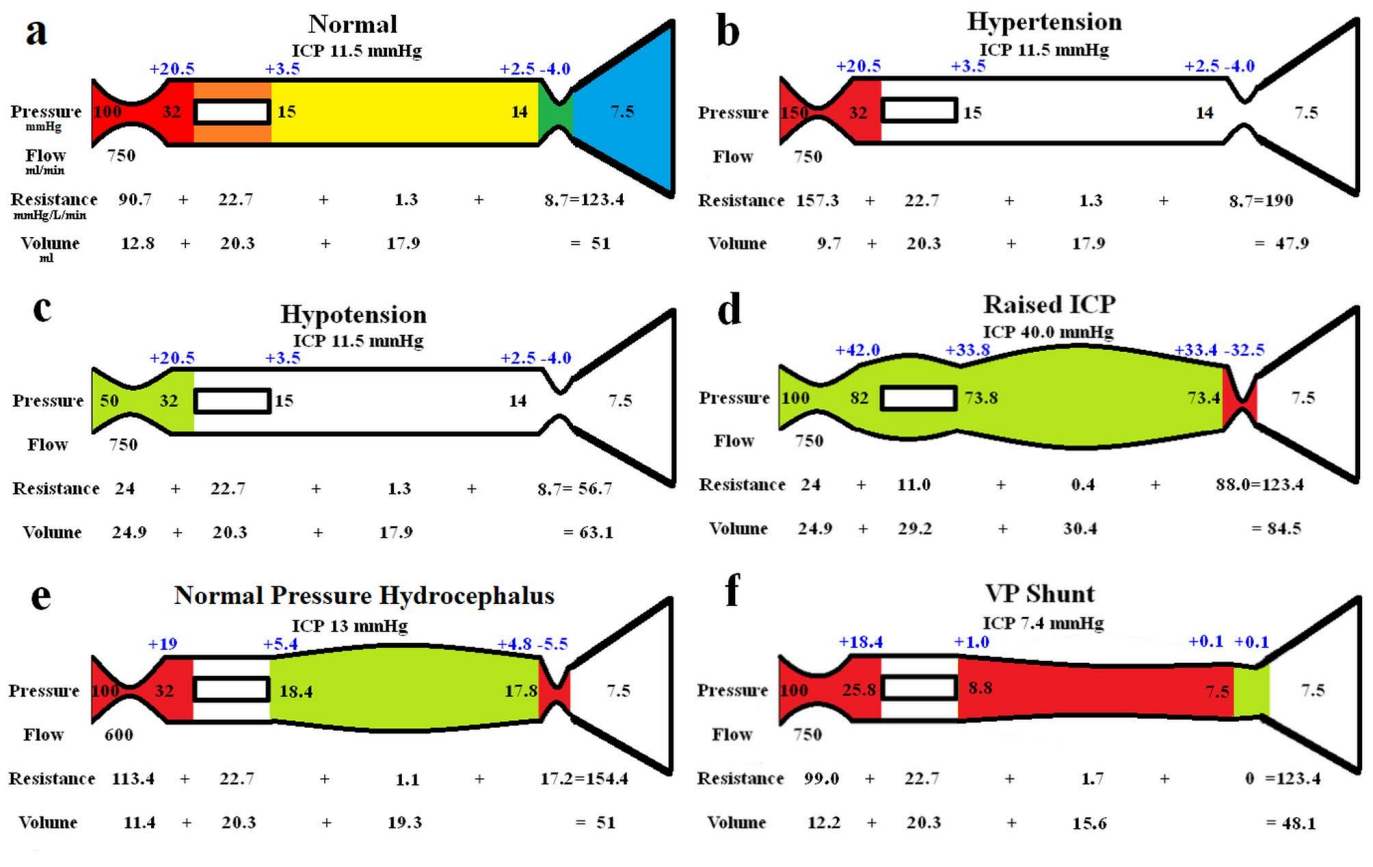
Results of modelling. (**a**) Depicts the normal findings. The red segment is the arterial, orange the capillary, yellow the veins, green the outflow cuff and blue the venous sinus. The vascular pressures are shown within the vessels. The blue numbers are the transmural pressures at each site. The resistances and volumes for each segment are shown below the vessel. (**b**) Shows the findings in hypertension with the red area indicating an increase in resistance in the arteries. (**c**) shows the findings in hypotension with the green area highlighting a reduction in resistance in the arteries. (**d**) Shows the findings in raised ICP with increased resistance in the outflow cuff and reduced elsewhere. Note the major resistance moves from the arteries to the outflow cuff. (**e**) shows the findings in normal pressure hydrocephalus. It should be noted that the changes are a mixture of 1b and 1c combined. (**f**) shows the findings following shunt insertion with decreased resistance in the outflow cuff. Note the reduction in arterial resistance as compared to NPH.

The modelling findings are summarized in Fig. 1. The five segments modelled are shown in Fig. 1a, with the arterial segment shown in red, the capillaries in orange, the veins in yellow, the outflow cuff in green and the sinus in blue. The pressures obtained from the literature have been appended to the beginning and end of each vascular segment. Given the arterial inflow volume passes through each segment sequentially, the resistance of each segment can be calculated using Eq. (2). These resistances are appended below the vessels in Fig. 1. The normal CBV values for each segment and the total CBV has been obtained from the literature and is shown below the resistances. The blue numbers represent the transmural pressure gradients between the pressure at the beginning and end of each capacitance vessel segment and the ICP and are obtained by subtraction. Figure 1b-f represent the effects of the differing alterations in perfusion pressure. In these figures, the red segments represent the areas of increased resistance compared to the normal findings and the green represent reduced resistance.

In Fig. 1b, the upper limit of autoregulation from hypertension (i.e., an arterial inflow pressure of 150 mmHg and a preserved blood flow rate) have been modelled. It is envisaged that the ICP will be unchanged in the steady state, with the CSF production making up for any loss of total intracranial volume over time as required by the Monroe-Kellie Doctrine. As described in the tube laws, the pressure in the capillaries, veins and cuff segments are unchanged with successful autoregulation. Therefore, the resistances and volumes of these segments will be unchanged from the normal values. Note, the total resistance across the system increases due to the larger pressure drop but preserved flow volume using Eq. (2). Utilizing Eq. (3) it can be seen that all of the increased resistance must be across the arteries. The 73.4% increase in arterial resistance equates to a 24% reduction in the arteriolar blood volume using Eq. (9). This leads to a reduction in the total CBV of 6.1% overall.

Figure 1c is at the opposite end of the autoregulation spectrum (i.e. hypotension) and is modeled with an inflow pressure of 50 mmHg and a preserved flow rate of 750 ml/min. Similar to the last model, it is envisaged that the ICP in the long term will revert to normal once any increase in volume is dissipated by the normal CSF absorption mechanism reducing the CSF volume. Again, the capillary, venous and cuff pressures are unchanged so the resistances and vascular volumes are also unchanged. The total resistance across all segments must be reduced because the pressure drop is less, but the flow rate is preserved and all of this resistance change must be within the arterial segment. The 73.5% reduction in arterial resistance equates to a 94.5% increase in arterial volume using Eq. (9). Thus the total change in the CBV from hypotension is an increase of 23.7%.

The final autoregulation case modelled is an increased ICP of 40 mmHg (Fig. 1d). The inflow pressure and flow rate are preserved, so the total resistance across all segments must be normal. By definition, because the limit of arterial autoregulation is reached, the arterial segment must be maximally dilated as in Fig. 1c, with the same reduction in resistance and the same volume change. However, unlike in 1c, the arterial inflow pressure is normal, so using the reduced resistance across this segment and the preserved flow rate, the pressure drop can be calculated using Eq. (2). The resulting pre-capillary bed pressure is significantly elevated and the transmural pressure here is more than doubled. In the capillary tube laws as already discussed, we noted that a fully dilated arterial segment with a normal arterial inflow pressure leads to a 44% increase in capillary volume. Using Eq. (9) a 44% increase in capillary volume equates to a 51.5% reduction in the resistance for this segment. Therefore, because the capillary resistance is reduced, Eq. (2) indicates the pre-venous pressure and resulting transmural pressures will also be grossly elevated. The tube law for the veins suggests an increased ICP will increase the veins by up to 70%, thus ensuring a reduction in venous resistance using Eq. (2) would be 69%. As the resistances in the arteries, capillaries and veins have all dropped, in order for Eq. (3) to be resolved, the cuff resistance must be increased 10 fold. The total CBV for this model has increased by 66% above the normal figure.

The modelling of NPH is shown in Fig. 1e. As discussed, the average arterial inflow is reduced by 20% in NPH, giving a flow rate of 600 ml/min. The CSF pressure is slightly increased in NPH being 13 mmHg and the CSF formation rate is reduced at 0.25 ml/min^11^. In the Dutch normal pressure hydrocephalus study, the upper limit of normal for the R_out_ was 18 mmHg/ml/min, and a good outcome from treatment was noted in individuals with an average R_out_ of 24 mmHg/ml/min^12^. Placing these figures into Eq. (1) gives a venous sinus pressure of between 7.0 and 8.5 mmHg i.e. it is normal. Therefore, the total vascular resistance must be increased by 31 mmHg/L/min using Eq. (2). Four groups have measured the CBF and CBV in NPH patients and despite finding a significant reduction in global CBF of up to 43%, all four found the global CBV to be within the normal range^13–16^. This is an apparent paradox, with one group pointing out that if the reduced flow were due to an arterial constriction, the CBV should be reduced by 18%^15^. Figure 1d predicts that if the increase in resistance resided within the venous outflow, then the CBV should be significantly increased. Therefore, the only way to reconcile an increased resistance, but a normal global CBV, is for the constriction of the arteries to be exactly balanced by the dilatation of the veins. Given the reduction in blood flow, the capillaries should have a close to normal TMP and therefore be unchanged in size. Thus, any decrease in arterial volume must be matched by an increase in venous volume exactly. This significantly constrains the model because a reduction in volume in the arteries will alter their resistance by a known amount using Eq. (9) and also the balanced volume change will alter the venous resistance by a known amount using the same equation. In addition, the increase in volume of the veins will require a change in the vein TMP as predicted by Eq. (10). As the venous outflow pressure can be calculated by subtracting the vein TMP from the ICP, the venous volume also sets the pressure drop across the outflow cuff and therefore, its underlying resistance. Initial modelling in NPH showed that a large change in arterial and venous volume made the resistances overshoot the target of 154.4 mmHg/L/min, a low value did the opposite. Solving the equations by an iterative method revealed that the optimum change in volume for both was a decrease in arterial area and increase in venous area of 1.35 ml, giving the resistances and pressures as noted in Fig. 1e.

Finally, the effect of placing a shunt tube was modelled in Fig. 1f. In the 6 most commonly inserted medium or normal pressure shunt valves, the average differential pressure in the horizontal position at a flow rate of 20 ml/hr (a normal rate) was 10 cmH_2_O^17^, which is 7.4 mmHg. In three studies, inserting a shunt tube returned the CBF back to the normal range^4,7,18^. Therefore, as the pressure drop across the entire system is normal and the flow has returned to normal post shunt, the total resistance also returns to normal. As the shunt sets the ICP to just below the sinus pressure, the TMP across the outflow cuff becomes positive and therefore it fully dilates, reducing the resistance of this short segment to effectively zero. The TMP of the veins are also reduced to close to zero reducing their volume by 12% using Eq. (10). The average TMP for the capillaries is reduced slightly so they do not change in volume or resistance. In order for Eq. (3) to balance, the arteries must dilate and reduce their resistance by 14.4 mmHg/L/min compared to NPH.

## Discussion

Cerebral perfusion depends on the pressure across the vasculature. The cerebral perfusion pressure is dependent on the mean arterial pressure minus the ICP^19^. The brain does not tolerate hypo- or hyper-perfusion, therefore, the maintenance of a constant flow over a range of pressures is achieved by autoregulation^19^. The maintenance of this flow is a myogenic mechanism, producing a constriction or relaxation of the vascular smooth muscle in the arterioles^19^. It has been noted that an increase in perfusion pressure from an increase in arterial pressure or reduction in the ICP constricts the arterioles and reduces the CBV^19^ (see Fig. 1b). Conversely, any reduction in cerebral perfusion pressure from a reduction in arterial pressure or increase in ICP dilates the vessels, increasing the CBV^19^ (see Fig. 1c, d). These changes have been directly visualised in animal models. Fog showed rapid changes in pial arteries and arterioles in cats with manipulation of the arterial pressure, a rise in pressure contracted the vessels and a reduction caused an increase in size^20^. In anesthetized cats using direct observation through a closed window, the pial arteries dilated between 30 and 55% in area with an increase in ICP to 40 mmHg^21^. On the venous side, in pigs, a 10 mmHg increase in ICP dilated the cortical veins by 33% in area and a 20% increase in ICP dilated the veins by 57%^22^. In humans, autoregulation maintains the CBF for an arterial pressure range between 50 and 150 mmHg^23^ and for an ICP of up to 40 mmHg^24^. Therefore these are the limits which have been modelled in the study.

One may note that the TMP across the wall of the outflow cuff is normally negative and becomes more so with an elevation in ICP (see Fig. 1a-e). It has been said the flow through a vein will cease once the transmural pressure becomes negative because the vein will be fully collapsed^25^. Clearly this cannot be correct. Direct modelling of veins obtained from dogs has shown a positive transmural pressure maintains a rounded configuration to the vein^26^. A negative transmural pressure first transforms the vein into an oval^26^. With a further reduction in transmural pressure, the long sides of the oval pinch in and contact each other^26^. The resulting dumbbell configuration leaves two parallel channels of a smaller cross-section, which greatly resist further compression due to the significantly increased bending forces required^26^. Therefore, complete collapse is not a feature of the venous system until the TMP approaches the arterial pressure.

There are many assumptions inherent in lumped parameter modelling. We can test the modelling we have performed by comparing the outcomes predicted by the model with the literature. In Fig. 1b, the arterial pressure is increased to the autoregulation limit and the resistance required to maintain a normal flow rate is increased by 66.6 mmHg/L/min. Using the assumptions from this model, the decrease in total CBV is estimated to be 6.1%. In humans, hypocapnia induced arterial constriction caused a 6% reduction in CBV in one study^27^ and 7.2% in another^28^. These are similar to our findings. In Fig. 1c the limit of autoregulation was tested by reducing the inflow pressure to 50 mmHg. The reduction in resistance required gave a calculated increase in total CBV of 23.7%. Grubb et al. reduced the arterial pressure in Rhesus monkeys to their limit of autoregulation and the CBV increased by 25% compared to the normotensive animals^29^, again this is similar to our findings. In the same primate model, increasing the ICP from 8.6 to 71 mmHg increased the CBV by 66%, increasing the ICP further to 94 mmHg did not alter the CBV but did reduce the CBF^30^. This indicates the autoregulation limit was transgressed at 94 mmHg and the elastic limit for the venous distension was also probably reached. These findings are identical to our findings that raising the ICP to the limit of autoregulation increases the CBV by 66%. Thus, we would contend that our modelling is accurate enough for our current purposes.

It has been shown that NPH is associated with a reversible 20% reduction in CBF. The cause of this reduction in flow has remained a topic for discussion. The reversible nature of the blood flow reduction, following shunt insertion, indicates the cause is not due to irreversible arterial damage or loss. Mathew et al. suggested the dilatation of the ventricular system stretches the anterior cerebral arteries over the corpus callosum, thereby reducing the blood flow^14^. Ventricular dilatation has also been suggested to directly compress the capillary bed and the venous drainage within the parenchyma, limiting the flow^31^. Our modelling indicates that none of these suggestions can be correct. The collapse of the venous bed should start at the most distal point, where the venous pressure is the lowest, and move towards the arterial end as the ICP increases^32^. This is because the minimum transmural pressure always occurs where the venous pressure is lowest. In order for blood to flow, the pressure within the lumen must reduce with the distance from the arteries, so the distal end of the veins is where the collapse occurs. It has been shown the cortical veins are only emptied if the ICP approaches the arterial pressure^33^. The model for NPH (Fig. 1e) appears to be an amalgamation of the raised ICP and hypertension models (Fig. 1b and c combined).

A surprising prediction of the model is that a 37% increase in the TMP across the outflow cuff in NPH leads to a 98% increase in the resistance across this segment. However, this finding is similar to the findings of Portnoy et al. who by direct measurement, noted a 34% increase in transmural pressure across the outflow segment in dogs with hydrocephalus, produced a 102% increase in the pressure gradient from the veins to sinuses^34^. The model indicates a large increase in cuff resistance is required despite the minor 1.5 mmHg increase in CSF pressure. This is because to simultaneously balance the increase in venous volume to the decrease in arterial volume in NPH in our model, required an increase in venous transmural pressure of 3.8 mmHg to dilate the vein by this amount. This elevated venous pressure increased the pressure gradient across the cuff to the venous sinus and therefore the cuff resistance. It turned out the only way to satisfy both the criteria for a balanced change in arterial and venous volume and a total vascular resistance of 154.4 mmHg/l/min (including the recalculated cuff resistance) was the result as shown in Fig. 1e. The reason why the apparent cuff resistance increases by so much in NPH is not elucidated by the current model. The current model is a non-pulsatile, steady state model. In other studies, we have speculated that an increase in cuff resistance of this amount may be a function of changes in dynamic venous compression from an increase in CSF pulse pressure in hydrocephalus altering the venous impedance pumping^35^. Interested readers are directed to this paper but further discussion of this topic is outside the remit of the current study. A further prediction of the model is that the transmural pressure across the vein wall increases by 92%. Such an increase in TMP should make the vein walls much stiffer than normal. The insertion of the shunt reduced the transmural pressure to approximately zero which should make the veins floppier than normal. Indeed, modelling using dog inferior vena cava has shown that the maximal compliance of veins occurs at close to a neutral transmural pressure. Increasing the transmural pressure by 4 mmHg reduces the compliance of the vein by 80%^26^. In NPH, the CSF pulse pressure is increased by 6–8 times normal^36^, meaning there is ample pulse pressure to rhythmically compress the veins. However, in NPH, the cortical vein pulsatility index is reduced by 33% compared to controls (indicating the veins are very stiff) and increased by 90% after shunting, indicating they are made very floppy^37^ correlating with the models prediction.

**Fig. 2:**
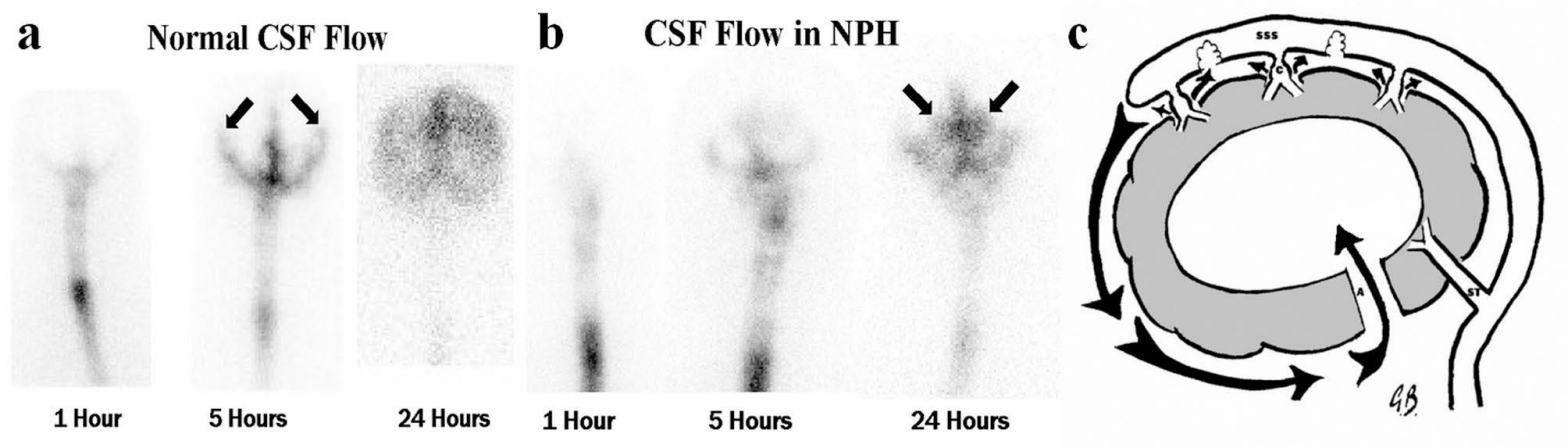
Flow of CSF in NPH. (**a**) Normal study radionuclide cisternogram showing the tracer diffusing and passing over the cortex (arrows) with no ventricular reflux. (**b**) A patient with NPH. Anterior radionuclide cisternogram showing reflux of the tracer into the ventricles (arrows) with no passage over the cortex. (**c**) A diagram of CSF flow in NPH. There is increased production of interstitial fluid over the vertex, which passes out via the perivenous spaces beside the cortical veins (C) and fails to be absorbed into the sagittal sinus (SSS). The fluid passes down and around the brain to reflux into the aqueduct (A). The CSF is absorbed through the wall of the ventricle. This figure from reference^37^ was reproduced with permission from Springer Nature.

The model suggests that the reduction in blood flow comes about from an increase in resistance. However, of the 31 mmHg/L/min increase in resistance required, 73% comes from the arterial segment and only 27% from the venous. As the increase in arterial resistance is largely reversible with a shunt, it would suggest that the brain is choosing to remain ischemic. This indicates there must be an ulterior motive behind this response. In a kaolin dog model of hydrocephalus, the initial hydrocephalus phase was associated with an elevation in CSF pressure. In the chronic phase, the CSF pressure returned to normal^38^, similar to NPH. Could it be that the arterial constriction in NPH is an attempt to limit the rise in ICP in chronic hydrocephalus? With blockage of the CSF outflow, the elevation in ICP is very dependent on the CSF production rate. The CSF production rate in NPH is reduced by 17% (0.25 ml/min cf. 0.3 ml/min). 70% of the CSF production comes from the choroid plexus, 18% from the capillaries with 12% from endogenous water production^39^. The endogenous water production comes about from the metabolism of glucose. There is a reduction in brain metabolism from the ischemia in NPH, partly accounting for the reduction in the CSF formation rate. Could the remainder of this reduction be from CSF absorption through the capillary walls? Hladky and Barrand argue that net absorption of CSF across an intact blood-brain barrier is not sustainable, regardless of the hydrostatic pressure difference, because the salt would be left behind and a rapid increase in osmotic pressure would negate the hydrostatic pressure difference within minutes^40^. However, in human hydrocephalus, the capillary walls show blood brain barrier dysfunction, with increased vesicular and vacuolar transport, open inter-endothelial junctions, thin and fragmented basement membranes, and discontinuous perivascular astrocytic end-feet^41^. The model predicts the average capillary bed transmural pressure gradient is close to normal in NPH and by increasing the leakiness of the capillaries, the CSF production would tend to increase and not decrease. However, despite the fact that the global CBV is normal in NPH, the white matter just beneath the ependyma of the ventricles is different. In NPH, the white matter immediately beneath the ependyma showed a reduction in CBV of 31% compared to controls, but the more superficial white matter had a normal CBV^42^. The CBF in the subependymal white matter is also much more ischemic than the remainder of the brain. The CBF was 66% lower in the periventricular tissue compared with more superficial tissue, with no such gradient being seen in controls^2^. Such a low blood flow with a reduced CBV can only come about by arterial rather than venous constriction. This severe arterial constriction would significantly reduce the capillary bed pressure in this segment and increase the CSF absorption through this route via the leaky blood brain barrier as noted. The predicted slightly increased CSF production over the vertex, together with the blockage of the arachnoid granulations and the absorption of CSF via the ventricular ependymal, would reverse the normal CSF flow direction (see Fig. 2). It has been noted all forms of communicating hydrocephalus are associated with an apparent blockage to CSF flow at the vertex and also ventricular reflux of fluid^43^, confirming this prediction. Following injection of a radiolabelled tracer into the subarachnoid space in the lumbar region, normally there is passage of the tracer up the spinal canal, around the convexities and toward the vertex (Fig. 2a). This occurs largely due to the effect of the second law of thermodynamics, ensuring that a concentration gradient will be dissipated due to diffusion, pulsatile mixing of fluid plus any net flow occurring. The lack of any mixing of tracer into the ventricles suggests there must be net flow from the ventricles to oppose the increase in entropy the second law requires. In Fig. 2b, in NPH, the diffusion of the tracer over the vertex is opposed, suggesting a net CSF production and the reflux into the ventricles would suggests a reversal of flow into the ventricles. Figure 2c is the diagrammatic representation of this effect.

The prediction of increased venous pressure in the veins over the cortex but reduced venous pressure around the ventricles, would tend to make the outer brain parenchyma stiffer and the inner brain floppier^44^ because the veins act as a hydraulic skeleton. This very finding has been noted in magnetic resonance elastography studies of NPH and is specific to this disease^45^. The modelling of NPH shows an 8% increase in the parenchymal vein size pre-shunt and a reduction in these veins of 29% post-shunt. The glymphatic fluid flow in the brain is hypothesised to occur by CSF inflow into the arterial perivascular spaces and outflow via the perivenous spaces^46^. Dilatation of the veins would be expected to narrow the venous perivascular spaces and impede the glymphatic outflow^47^. MRI imaging techniques confirm there is glymphatic obstruction in NPH and there is a significant improvement in glymphatic flow following shunt insertion^48^.

The modelling we have provided is only as good as the assumptions which must be made to accomplish it. For example, Poiseuille’s equation requires flow through a thin, rigid, circular tube of a Newtonian fluid, without turbulence. To the degree that these assumptions hold, the findings would be accurate. However, despite its limitations, this equation is commonly used in modelling the vasculature. The second significant assumption is that Davson’s equation accurately predicts the venous pressure in NPH. In dog models of hydrocephalus, in the acute phase, the venous pressure actually increases. In the chronic phase (similar to NPH) the venous pressure reduces together with the CSF pressure but at a lower rate than the CSF. Therefore, the venous pressure remains elevated compared to the ICP, with a loss of the pressure gradient across the arachnoid granulations reducing CSF absorption^38^. A similar finding may occur in humans. In adults with chronic hydrocephalus averaging 45 years old, the combined right and left transverse sinus areas were reduced by 38% with the flow through the sinuses being reduced by 24% compared to controls^49^. The estimated sinus pressure was increased to 10 mmHg due to the greater effect of the area reduction compared to flow reduction^49^. A sensitivity analysis was performed to quantify the changes such an elevation in sinus pressure would bring to the model. The increased venous pressure would reduce the resistance of the outflow cuff and the pressure drop across it, but because the sinus pressure is increased, the venous pressures were little changed overall. The percentage of the change in total resistance as apportioned to the arteries increased from 73% in the original model to 87% in the model with elevated sinus pressure because the venous resistance dropped by a proportionally greater amount than the arterial resistance.

## Methods

### Equations

The study begins with Davson’s equation which relates the intracranial pressure (ICP) to the CSF formation rate, the CSF outflow resistance and the venous sinus pressure^50^.

1 $$\:ICP={FR}_{csf}\times\:{R}_{out}+{P}_{sss}$$

Where ICP is the intracranial pressure, FR_csf_ is the CSF formation rate, R_out_ is the CSF outflow resistance and P_sss_ is the pressure in the superior sagittal sinus. Next Ohms law for hydraulic circuits is required:

2 $$\:\varDelta\:P=Q\:\times\:\:R$$

Where ΔP is the pressure drop across a vascular segment, Q is the flow rate through the segment and R is the resistance. As resistances in series are directly additive the following can be derived:

3 $$\:{R}_{art}+{R}_{cap}+{R}_{ven}+{R}_{cuf}={R}_{tot}$$

where R_art_ is the arterial segment resistance, R_cap_ is the resistance of the capillaries, R_ven_ is the venous resistance, R_cuf_ is the resistance of the venous outflow cuff and R_tot_ is the total resistance for the entire vascular system. Poiseuille’s equation calculates the pressure drop across each of these segments:

4 $$\:\varDelta\:P=8\mu\:LQ/\pi\:{r}^{4}$$

where ΔP is the pressure drop, µ is the viscosity, L is the vessel length, Q is the fluid flow rate, π is the circle proportionality constant and r is the radius. Substituting Eq. (2) into (4) and eliminating Q from both sides gives an equation for the resistance in each segment:

5 $$\:R=8\mu\:L/\pi\:{r}^{4}$$

In this modeling study the viscosity, the length of each vessel segment, and π are constants, so it can be shown that a change in resistance for any segment depends only on a change in the vessel radius i.e.

6 $$\:\varDelta\:R=\varDelta\:{r}^{-4}$$

The volume of a vessel is given by the equation for a cylinder i.e.

7 $$\:V=L\pi\:{r}^{2}$$

where V is the volume, L is the vessel length and r is the radius of the vessel. Given L and π are constants for any given segment, the change in volume is dependent on the change in radius i.e.

8 $$\:\varDelta\:V=\varDelta\:{r}^{2}$$

Substituting Eq. (8) into Eq. (6) gives

9 $$\:\varDelta\:R=\varDelta\:{V}^{-2}$$

The final equation required relates the transmural pressure across a vessel to the vessel cross-sectional area^51^:

10 $$\:{P}_{tm}=\frac{4Eh}{{3R}_{o}}\left(1-\sqrt{\frac{{A}_{o}}{A}}\right)$$

where P_tm_ is the transmural pressure across the vessel wall (lumen pressure- CSF pressure), E is the circumferential Young’s modulus of the vessel wall, h is the wall thickness, R_o_ is the radius in the stress free state, A_o_ is the area in the stress free state and A is the area following the applied transmural pressure.

### Model input parameters

This study is based on a middle aged individual with a brain size of 1500 g. A normal global CBF is 50 ml/100 g/min^52^, giving a normal cerebral blood arterial inflow of 750 ml/min. The normal mean arterial inflow pressure is 100 mmHg^53^. The normal precapillary bed pressure is between 30 and 35 mmHg^54^ and has been designated to be 32 mmHg in this study. The end capillary pressure is estimated to be 15 mmHg^32^. The normal CSF pressure in middle age is 11.5 mmHg^55^ and the normal pressure gradient from the CSF to the superior sinus lumen is 4 mmHg^56^, giving a normal sinus pressure by subtraction of 7.5 mmHg^49^. The normal transmural pressure of the subarachnoid cortical veins has not been measured in humans. Using three separate methods to vary the ICP, Johnston and Rowan using baboons showed the cortical venous transmural pressure (TMP) for all three methods combined equaled the ICP plus 2.5 mmHg^57^. This suggests the venous transmural pressure for primates averages 2.5 mmHg. Using this figure for the model, it can be seen that the pre-venous outflow cuff pressure is 14 mmHg by addition of the TMP to the ICP.

The cerebral blood volume (CBV) in the grey matter is 4.6 ml/100 g and in the white matter it is 1.3 ml/100g^58^. The grey matter volume makes up 65% of the brain and with matter being 35%^59^ giving a weighted global average CBV of 3.4 ml/100 g. Thus, in a 1500 g brain, the total CBV would be 51 ml. In a review of 18 studies, Hua et al. found the arterial component of the CBV to be between 20 and 30%^60^. Therefore, 25% has been taken to be the figure for this study or 12.8 ml in total. This leaves the remaining 75% for the capacitance vessels, including the veins and capillaries or 38.2 ml. The estimated percentage of this latter figure being the capillary volume has varied significantly in several studies from 20%^61^, 52%^62^, 53%^63^, 59%^64^ and 72%^65^. Given the upper and lower figures are outliers, the middle figure of 53% has been chosen for this study, giving a total capillary blood volume of 20.3 ml and a total venous blood volume of 17.9 ml.

The normal CSF outflow resistance (R_out_) has been found to depend linearly with age, with the regression line being; 9.88 + 0.075 x Age^66^. This gives a normal R_out_ for a 45 year old of 13.3 mmHg/ml/min. The normal CSF formation rate is highest in children and young adults, being about 0.4 ml/min and decreases with age to about 50% of this value at age 70 years^67^. This indicates a normal CSF production rate in middle age is 0.3 mls/min. Thus, Davson’s Eq. (1) would confirm the normal pressure gradient across the wall of the venous sinus to be 4 mmHg in middle age (13.3 × 0.3) with a resulting normal sinus pressure of 7.5 mmHg.

### Vessel responses to transmural pressure variations

It is assumed that variations in the arterial resistance and volume in this model depend entirely on the arterial autoregulation and muscle tone and not the vessel transmural pressure. As the arterial pressure is always much higher than the ICP, the arterial transmural pressure will have no effect on the outcome of the current modelling study.

In the capillary bed, the vessels do not actively alter their diameter^23^, indicating they react purely to their transmural pressure. It has been said the capillary TMP will not be significantly altered by elevations or reductions in the arterial pressure whilst within the autoregulation zone of the perfusion pressure because a change in arterial resistance will balance the inflow pressure change^25^. This ensures the preservation of the correct balance of tissue exchanges to maintain water, gas and solutes^25^. To emphasize this fact, in a rat model, extreme hyperventilation decreased the PCO_2_ from 40 to 21.6 mmHg without affecting PO_2_, the capillary size was not significantly different to controls despite the expected arteriolar constriction^68^. However, in the opposite case, in rats made extremely hypercapnic secondary to hypoventilation, the PCO_2_ increased to 95.6 mmHg but PO_2_ was normal, the capillary diameter increased by 20% consistent with a 44% increase in volume compared to known control values^68^. Thus, a moderate reduction in inflow pressure does not change the capillary size but maximal dilatation of the arterioles associated with a preserved arterial inflow pressure will maximally increase the capillary transmural pressure and dilate these vessels by 44% increasing their volume.

**Fig. 3:**
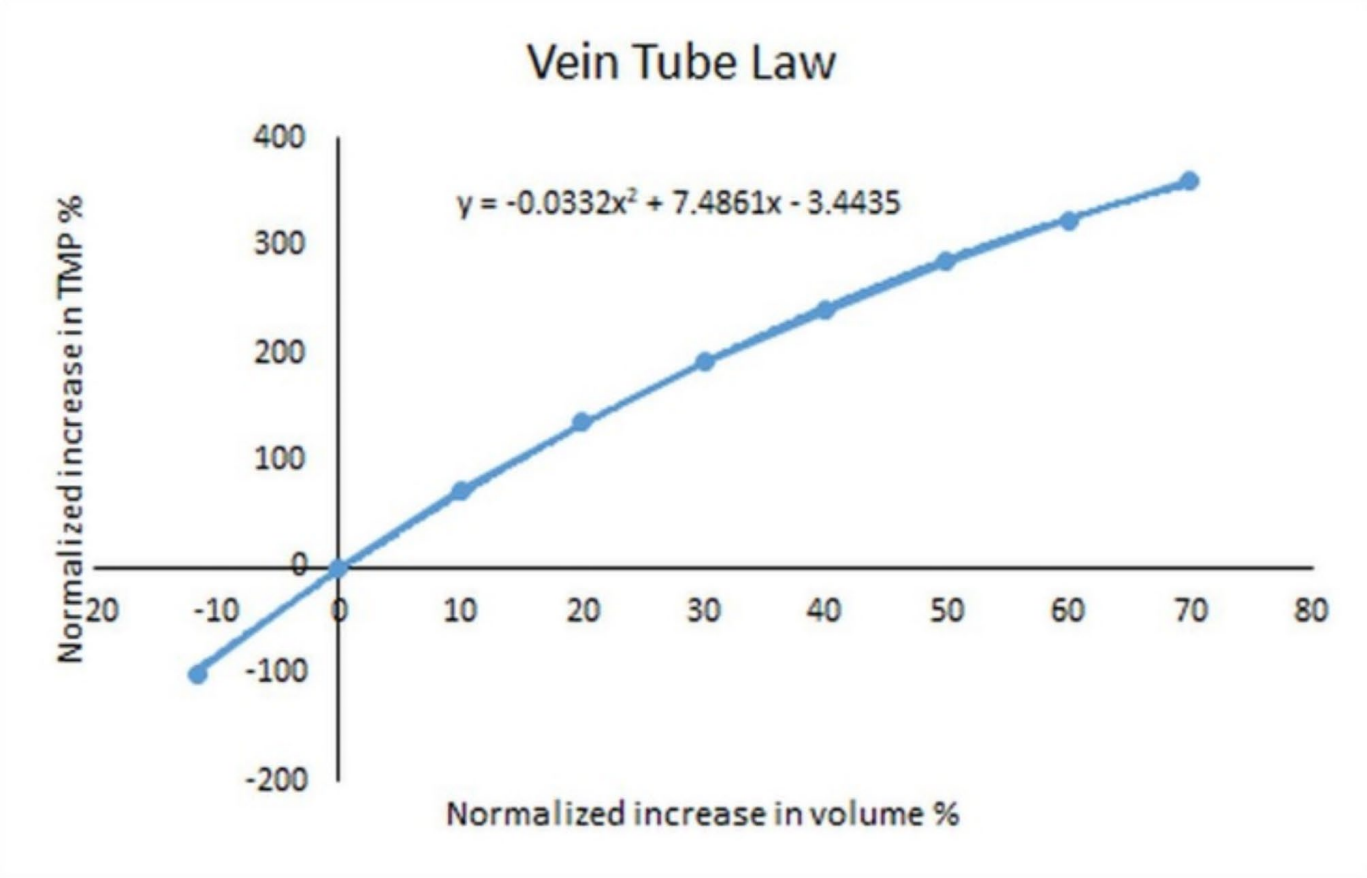
Relationship between the change in transmural pressure and volume of cortical veins. A graph of the change in volume of the cortical veins vs. the change in transmural venous pressure which was derived from Eq. (10).

Similar to the capillaries, the veins alter their size purely depending on their transmural pressures. Again, moderate changes in arterial pressure will alter the upstream vascular resistance which prevents increased or decreased pressures developing within the pial veins^69^. As will be discussed, the distal cortical veins contain a cuff which collapses under the ICP. This increases the upstream venous luminal pressure and the upstream veins will dilate due to an increase in their TMP. In human subjects, the bridging cortical veins show a mean diameter of 2.04 mm under normal intracranial pressure but increase to 2.65 mm under a significantly increased ICP, a 69% increase in cross-sectional area/ volume^70^. It is not envisaged that the range of ICPs modeled within this study will extend outside this range so an upper limit of venous dilatation of 70% in volume will be designated. The range of the required transmural pressures from 0 mmHg (the stress free state) to that pressure required to dilate the veins by 70% can be modeled using Eq. (10). In a previous modelling study, the elastic modulus in humans was estimated to be 0.163 MPa, the vein thickness was 0.044 mm and the resting state radius of the veins in the review was 1.55 mm^35^. As these values are not expected to change during changes in the transmural pressure, it can be seen that the change in the vessel area compared to the stress free state will vary with the transmural pressure. In addition, Eq. (7) indicates that, if the length of the venous segment is unchanged, then the change in area is equivalent to the change in volume. Therefore, the function relating the change in TMP vs. the change in volume for the cortical veins was investigated using Eq. (10). The results are summarized in Fig. 3. It can be seen that as expected, at a normal TMP, the volume change is unaltered from normal. Reducing the transmural pressure by 100% (i.e. reducing the normal TMP to zero) reduces the vein volume by 12% and a 70% increase in vein volume would require an increase in TMP of 362% above normal. The function is a quadratic equation which is appended to Fig. 3. At some point, the collagen within the vein walls will be fully stretched and therefore unable to expand any further (the elastic limit).

At the distal end of the cortical veins, as they join the sinus wall, the outflow cuff segment resides. Vignes et al. found using pathology specimens, that the bridging vein diameter remains constant along its subarachnoid course but dilates at a short section called the outflow cuff segment as it joins the sinus^71^. This segment has collagen which is aligned in a spiral fashion rather than longitudinally, as for the rest of the vein^71^. The collapse of this segment occurs physiologically, at the distal end of the cortical vein, and is passively modulated by the transmural pressure between the ICP and the sinus pressure, which is usually negative^25^. The segment is very short, and as it is mostly under a state of collapse with physiological ICPs, the change in volume from this segment will be ignored in this model. However, its resistance will be taken into consideration. The sagittal sinus pressure has been found to be independent of the ICP in the majority of individuals^72^ and so will be taken to be a constant in this study.

## Data Availability

All data generated or analysed during this study are avaliable within the original paper or on reasonable request to the coresponding author GAB.

## Abbreviations

CBF: Cerebral blood flow
CBV: Cerebral blood volume
CSF: Cerebrospinal fluid
g: Grams
ICP: Intracranial pressure
L: Liter
min: Minute
ml: Milliliter
MPa: Megapascal
mm: Millimeters
mmHg: Millimeters of mercury
PCO_2_: Partial pressure of carbon dioxide
PO_2_: Partial pressure of oxygen
R_out_: CSF outflow resistance
TMP: Transmural pressure
